# Suppression of the TGF-β signaling exacerbates degeneration of auditory neurons in kanamycin-induced ototoxicity in mice

**DOI:** 10.1038/s41598-024-61630-1

**Published:** 2024-05-13

**Authors:** Yoshihiro Nitta, Takaomi Kurioka, Sachiyo Mogi, Hajime Sano, Taku Yamashita

**Affiliations:** 1 Department of Otorhinolaryngology and Head and Neck Surgery, Kitasato University School of Medicine, 1-15-1 Kitasato, Minami-ku, Sagamihara, Kanagawa 252-0374 Japan; 2 Department of Otorhinolaryngology, National Defense Medical College, 3-2 Namiki, Tokorozawa, Saitama 359-8513 Japan; 3 School of Allied Health Sciences, Kitasato University, Kanagawa, Japan

**Keywords:** Transforming growth factor-β, Kanamycin, Ototoxicity, Spiral ganglion neuron, Cochlear inflammation, Cochlea, Hair cell, Inner ear

## Abstract

Transforming growth factor-β (TGF-β) signaling plays a significant role in multiple biological processes, including inflammation, immunity, and cell death. However, its specific impact on the cochlea remains unclear. In this study, we aimed to investigate the effects of TGF-β signaling suppression on auditory function and cochlear pathology in mice with kanamycin-induced ototoxicity. Kanamycin and furosemide (KM-FS) were systemically administered to 8-week-old C57/BL6 mice, followed by immediate topical application of a TGF-β receptor inhibitor (TGF-βRI) onto the round window membrane. Results showed significant TGF-β receptor upregulation in spiral ganglion neurons (SGNs) after KM-FA ototoxicity, whereas expression levels in the TGF-βRI treated group remained unchanged. Interestingly, despite no significant change in cochlear TGF-β expression after KM-FS ototoxicity, TGF-βRI treatment resulted in a significant decrease in TGF-β signaling. Regarding auditory function, TGF-βRI treatment offered no therapeutic effects on hearing thresholds and hair cell survival following KM-FS ototoxicity. However, SGN loss and macrophage infiltration were significantly increased with TGF-βRI treatment. These results imply that inhibition of TGF-β signaling after KM-FS ototoxicity promotes cochlear inflammation and SGN degeneration.

## Introduction

There are more than 100 types of ototoxic drugs, including aminoglycosides and platinum-based drugs. Each year, 180,000 doses of aminoglycosides are prescribed worldwide, which are crucial for treating infections and cystic fibrosis but are also reported to cause hearing loss in approximately 40% of patients^1^. Early detection and prevention of drug-induced hearing loss are paramount due to difficulties in recovery, even after drug cessation. Previous research has shown that kanamycin (KM) administration in mice resulted in hair cell (HC) and spiral ganglion neuron (SGN) loss in the cochlea^2^. KM has since been used in animal model studies for drug-induced cochlear damage. Moreover, since KM alone causes minor loss of HCs and SGNs, simultaneous administration of KM and furosemide (FS) exacerbated cochlear damage while improving survival due to the latter drug’s renal protective effect^3,4^.

Cochlear damage may be caused by diverse pathologies, including macrophage infiltration, inflammatory cytokine expression (TNF-α, IL-6, and IL-1), apoptosis, and fibrosis^5^. Reactive oxygen species (ROS) are known factors in ototoxicity- and noise-induced cochlear damage, resulting in neurodegeneration by caspase-mediated apoptosis^6,7^. Similarly, cochlear ROS has been reported to cause inflammation and inflammatory cytokine release (IL-6, TNF-α). Mitogen-activated protein kinase signaling, a key regulator of intracellular signaling pathways, is also involved in cochlear cell death^8^. Cochlear neuropathy is often considered a secondary change due to HC loss and the excessive glutamate release^9^. Aminoglycoside-induced HC loss predominantly affects the basal (high frequency) outer hair cells (OHCs), progressively extending to the inner hair cells (IHCs) and apical (low frequency) OHCs with increasing cumulative doses^10^. Recent evidence suggests that aminoglycosides enter the HCs through mechanoelectrical transduction (MET) channels at the OHCs tips, which accumulate and consequently cause damage^11^. However, the precise mechanisms underlying aminoglycoside-induced cochlear damage and its relation to clinical treatment remain elusive.

Transforming growth factor-β (TGF-β) is a cytokine that plays key roles in various biological processes, including inflammation, immunity, and cell death^12^. In particular, TGF-β signaling is involved in inducing inflammation, fibrosis, and cell proliferation in the kidney and liver, and its involvement in tumor cell proliferation has led to its investigation as an anticancer agent^13^. Notably, TGF-β immunostaining has been observed in the cochlea; however, it is unclear what role TGF-β signaling has in auditory physiology^14^. In addition, noise-induced cochlear TGF-β expression in the inner ear has been linked to hearing loss attenuation with TGF-β inhibitor administration and suppression of inflammatory cytokines and NADPH oxidase^15^. As such, there is potential for TGF-β signaling to become a novel treatment for drug-induced inner ear damage. However, despite these findings, the role of TGF-β signaling in ototoxicity-induced cochlear damage remains unknown. Therefore, in this study, we created an ototoxicity mouse model by administering KM and FS (KM-FS), examined the effects of TGF-β receptor inhibitor (TGF-βRI) administration on auditory function and cochlear pathology, and discussed the role of TGF-β signaling in drug-induced inner ear damage.

## Results

### Elevation of hearing thresholds and mortality rates following KM-FS administration

**Figure 1:**
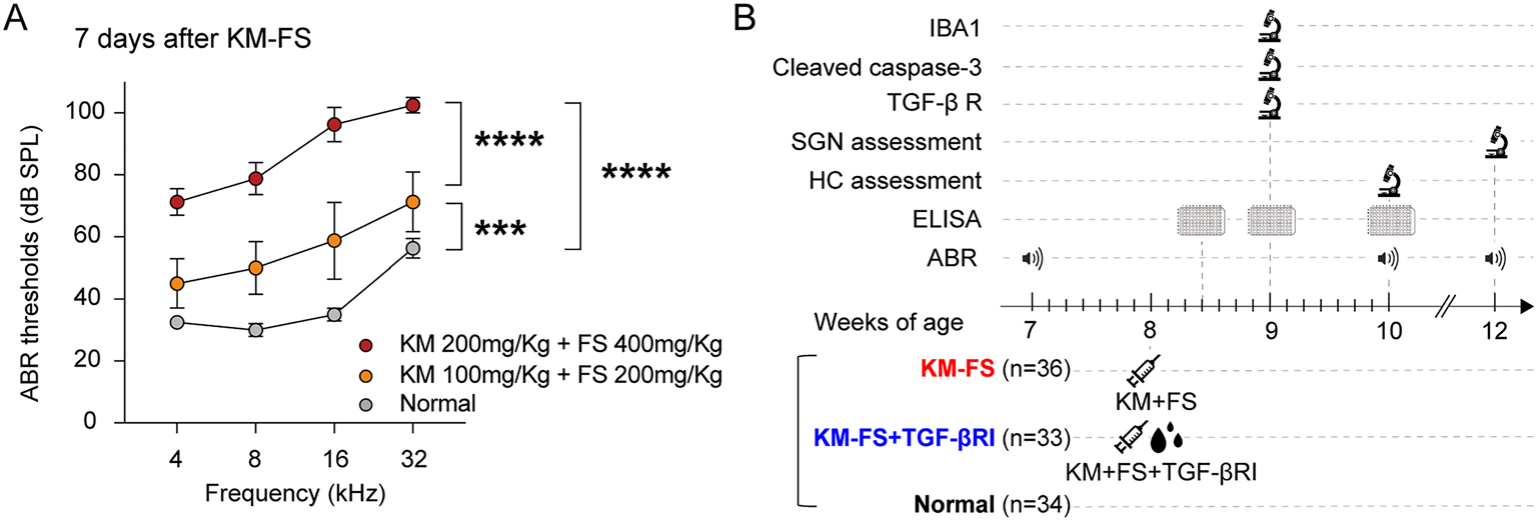
ABR thresholds following KM and FS administration experimental protocol. (**A**) ABR thresholds 7 days after KM and FS administration. ABR thresholds were significantly elevated in mice that received single doses of [KM 100 mg/Kg + FS 200 mg/Kg] and [KM 200 mg/Kg + FS 400 mg/Kg] compared to normal mice (****p < 0.0001, ***p < 0.001). (**B**) Animal groups and experimental procedures. ABR, auditory brainstem response; KM, kanamycin; FS, furosemide, Normal group: no ototoxic insults or surgical procedures were done, KM-FS group: left ears were exposed to KM-FS ototoxicity and DMSO topical application onto the RWM, KM-FS + TGF-βRI group: left ears were exposed to KM-FS ototoxicity and TGF-βRI topical application onto the RWM. HC, hair cell; RWM, round window membrane; SGN, spiral ganglion neuron; TGF-βRI, TGF-β receptor inhibitor.

To determine the protocols for creating the ototoxicity model, mice were intraperitoneally administered with single doses of KM-FS at various concentrations and examined for changes in hearing thresholds and mortality rates. Existing literature reports diverse dosages and administration regimens for KM-FS^16–18^. While higher doses of KM-FS can efficiently create ototoxicity models, they often lead to higher mortality rates due to systemic side effects, such as nephrotoxicity^4^. Initial administration of intraperitoneal KM (500 mg/Kg)-FS (500 mg/Kg) resulted in a high mortality rate of 81% (9/11 mice). Doses were lowered to KM (200 mg/Kg)-FS (400 mg/Kg) and KM (100 mg/Kg)-FS (200 mg/Kg), resulting in a mortality rate of 0% (0/8 mice). At 7 days after KM-FS administration, hearing threshold measurements via auditory brainstem response (ABR) demonstrated significant dose-dependent elevations (Fig. 1A). Notably, significantly higher ABR thresholds were observed in the KM (200 mg/Kg)-FS (400 mg/Kg) group compared to that in the normal and KM (100 mg/Kg)-FS (200 mg/Kg) groups. To minimize lethality in subsequent experiments, we used doses of KM (200 mg/kg)-FS (400 mg/kg), which have previously been demonstrated to safely create a mouse model of severe ototoxic cochlear damage^16^.

A total of 120 mice were assigned to the following groups for the investigation of TGF-β signaling (Fig. 1B): (1) the Normal mice group (n = 34 ears), where no ototoxic insults or surgical procedures were performed; (2) the KM-FS mice group (n = 36 ears), where systemic KM-FS ototoxicity was followed by sham surgery in which DMSO was topically applied to the round window membrane (RWM) of the left ear; and (3) the KM-FS + TGF-βRI mice group (n = 33 ears), where systemic KM-FS ototoxicity was followed by topical TGF-βRI application onto the RWM of the left ear. The sham surgery was performed by placing a gelatin sponge soaked in DMSO, the solvent for the TGF-βRI, directly above the RWM. The lowered KM-FS dose regime successfully decreased lethality; of the 86 mice administered KM (200 mg/Kg)-FS (400 mg/Kg), 17 died during the study period, resulting in a mortality rate of 19.8% (17/86). These mice were excluded from further analyses. No obvious signs of balance disorders, including instability or shakiness, were detected in any of the mice after KM-FS treatment.

### Changes in cochlear TGF-β expression following KM-FS administration

**Figure 2:**
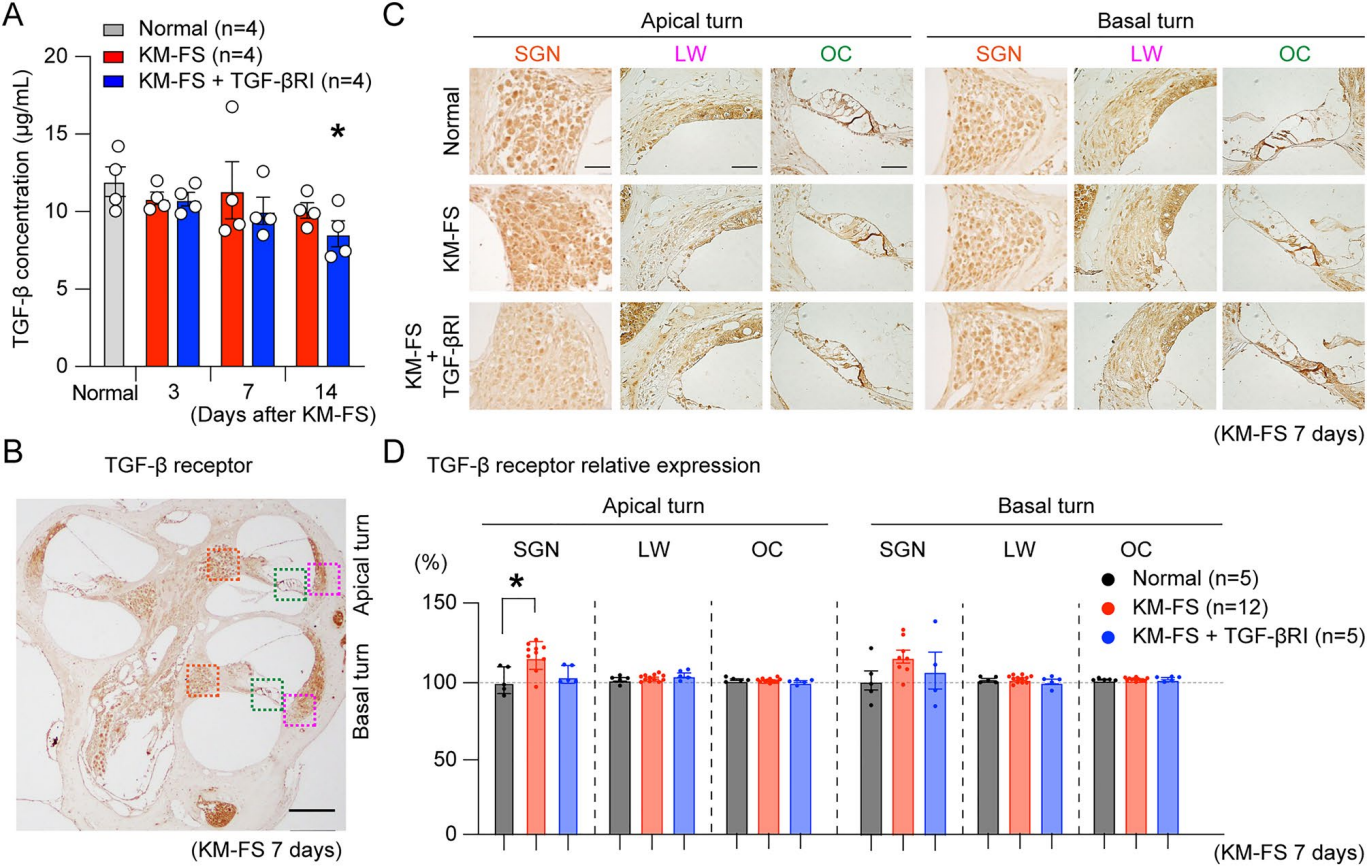
Quantification of cochlear TGF-β and TGF-β receptor expression. (**A**) Changes in cochlear TGF-β expression before (normal), 3, 7, and 14 days after KM-FS administration. TGF-β levels were significantly decreased in the KM-FS + TGF-βRI group after 14 days of KM-FS administration compared to that in the normal group. (**B**) Cochlear TGF-β receptor expression 7 days after KM-FS administration. TGF-β receptor expression was abundant in the SGN (orange dotted line), LW (pink dotted line), and OC (green dotted line). Scale bar indicates 200 μm. (**C**) Representative immunohistochemistry of TGF-β receptor expression in the SGN, LW, and OC in cochlear apical and basal turns 7 days after KM-FS administration. Scale bar indicates 20 μm. (**D**) Quantitative evaluation of TGF-β receptor expression. The expression was normalized to the values in each normal group. In the SGN of the cochlear apical turns, TGF-β receptor expression was significantly elevated in the KM-FS group compared to that in the normal group.KM, kanamycin; FS, furosemide; TGF-βRI, TGF-β receptor inhibitor; *p < 0.05; LW, lateral wall; OC, organ of Corti; SGN, spiral ganglion neuron.

To elucidate the role of TGF-β signaling in KM-FS-induced cochlear damage, we quantitatively assessed the changes in cochlear TGF-β expression levels before, 3, 7, and 14 days after KM-FS administration using ELISA assays (n = 4 ears for each time point). Although TGF-β expression levels did not change significantly at any time point in the KM-FS group, TGF-β expression levels were significantly lower in the KM-FS + TGF-βRI group than that in the normal group at 14 days after administration (*p* < 0.05, Fig. 2A).

### Assessment of TGF-β receptor expression

To investigate the role of cochlear TGF-β receptor expression after KM-FS administration, we performed immunohistochemistry to localize and quantify TGF-β receptor expression in the cochlea. Studies have shown that the expression of TGF-β-related genes was elevated in mouse cochleae 4 h after acoustic exposure, with persistence of elevated SMAD2/3 signaling (a downstream signal of TGF-β) 14 days later^15^. Thus, we evaluated these expressions at 7 days after KM-FS administration.

Robust TGF-β receptor expression was observed in the lateral wall (LW), organ of Corti (OC), and SGNs (Fig. 2B). Relative comparisons of TGF-β receptor expression were also performed on each group (Fig. 2C). In the LW and OC, no significant changes in the cochlear apical and basal turns were observed across all groups (*p* > 0.05, Fig. 2D). However, in the SGNs of the cochlear apical turn, TGF-β receptor expression was significantly increased in the KM-FS group compared to that in the normal group (*p* < 0.05), whereas expression levels were similar between the KM-FS + TGF-βRI and normal groups, indicating that the topical TGF-βRI application successfully decreased TGF-β expression in the cochlea. This was similarly observed in the SGNs of the cochlear basal turn.

### Assessment of ABR thresholds

**Figure 3:**
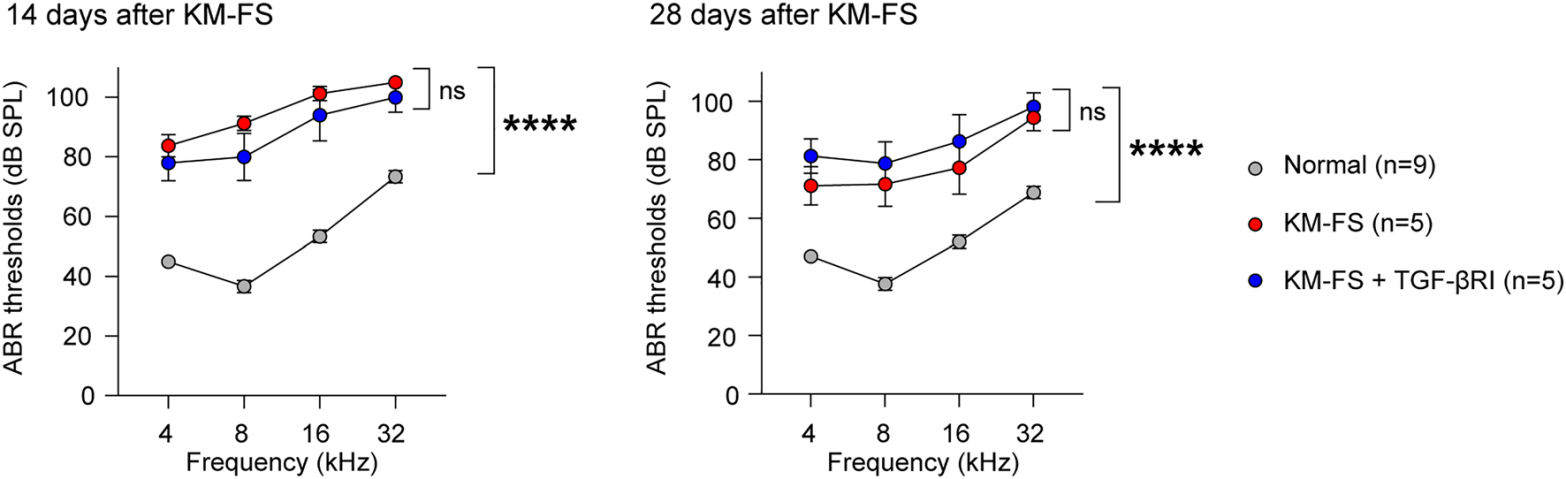
ABR thresholds at 14 and 28 days after KM-FS administration. At 14 and 28 days after KM-FS administration, ABR thresholds were significantly elevated in the KM-FS and KM-FS + TGF-βRI groups compared to that in the normal group. No significant differences in ABR threshold were observed between the KM-FS and KM-FS + TGF-βRI groups. ABR, auditory brainstem response; FS, furosemide; KM, kanamycin; SPL, sound pressure level; TGF-βRI, TGF-β receptor inhibitor. ****p < 0.0001.

ABR assessment performed at 7 weeks of age, just prior to KM-FS administration showed no significant differences in the hearing thresholds across groups (Supplementary Fig. S1). At 14 and 28 days after KM-FS administration, significant elevation of ABR thresholds were observed in the KM-FS (n = 5 ears) and the KM-FS + TGF-βRI groups (n = 5 ears) compared to that in the normal group (n = 9 ears) (two-way analysis of variance (ANOVA), KM-FS *vs.* normal, *p* < 0.0001; KM-FS + TGF-βRI *vs.* normal, *p* < 0.0001; Fig. 3). Conversely, no significant differences were observed between the KM-FS and KM-FS + TGF-βRI groups (two-way ANOVA, KM-FS *vs.* KM-FS + TGF-βRI, *p* > 0.05), indicating that topical TGF-βRI application had no therapeutic effect on KM-FS-induced hearing loss. Regarding the effects of RWM surgery on hearing thresholds in KM-FS mice, we performed an additional analysis comparing ABR thresholds between KM-FS mice with and without RWM surgery. There was no significant difference in ABR thresholds between the sham surgery left ears and non-surgery right ears of KM-FS mice (two-way ANOVA, *p* > 0.05, Supplementary Fig. S2), indicating RWM surgery does not affect the hearing thresholds following the KM-FS administration. In addition, regarding the contralateral ears of KM-FS + TGF-βRI mice, there was no significant difference in ABR thresholds in the contralateral ear between the KM-FS and KM-FS + TGF-βRI groups at 28 days after ototoxicity (two-way ANOVA, *p* > 0.05, Supplementary Fig. S3), indicating that it is unlikely that TGF-βRI crossed over to the contralateral ear.

Measurement of the peak 1 amplitude at 80 dB SPL stimulation 28 days after KM-FS administration revealed a significant difference among the three groups (two-way ANOVA, *p* = 0.0003). A significant decrease in the ABR peak 1 amplitude was observed in the KM-FS + TGF-βRI group compared to the normal group (two-way ANOVA, KM-FS + TGF-βRI *vs.* normal, *p* < 0.001, Supplementary Fig. S4). The ABR peak 1 amplitude is determined by cochlear nerve functions*,* and these decreases suggest cochlear nerve degeneration. It has been reported that loss of half of the cochlear nerve, resulting in a 50% reduction in response amplitude, does not affect the ABR threshold^19^; thus, a significant reduction in amplitude in the KM-FS + TGF-βRI groups may indicate a significant loss of SGN in the apical turn.

### Assessment of HC survival

To evaluate HC survival, immunostaining with Myosin7a and Phalloidin-iFluor 488 Reagent was performed 14 days after KM-FS administration, following a previous report that observed HC loss at this time point^3^.

**Figure 4:**
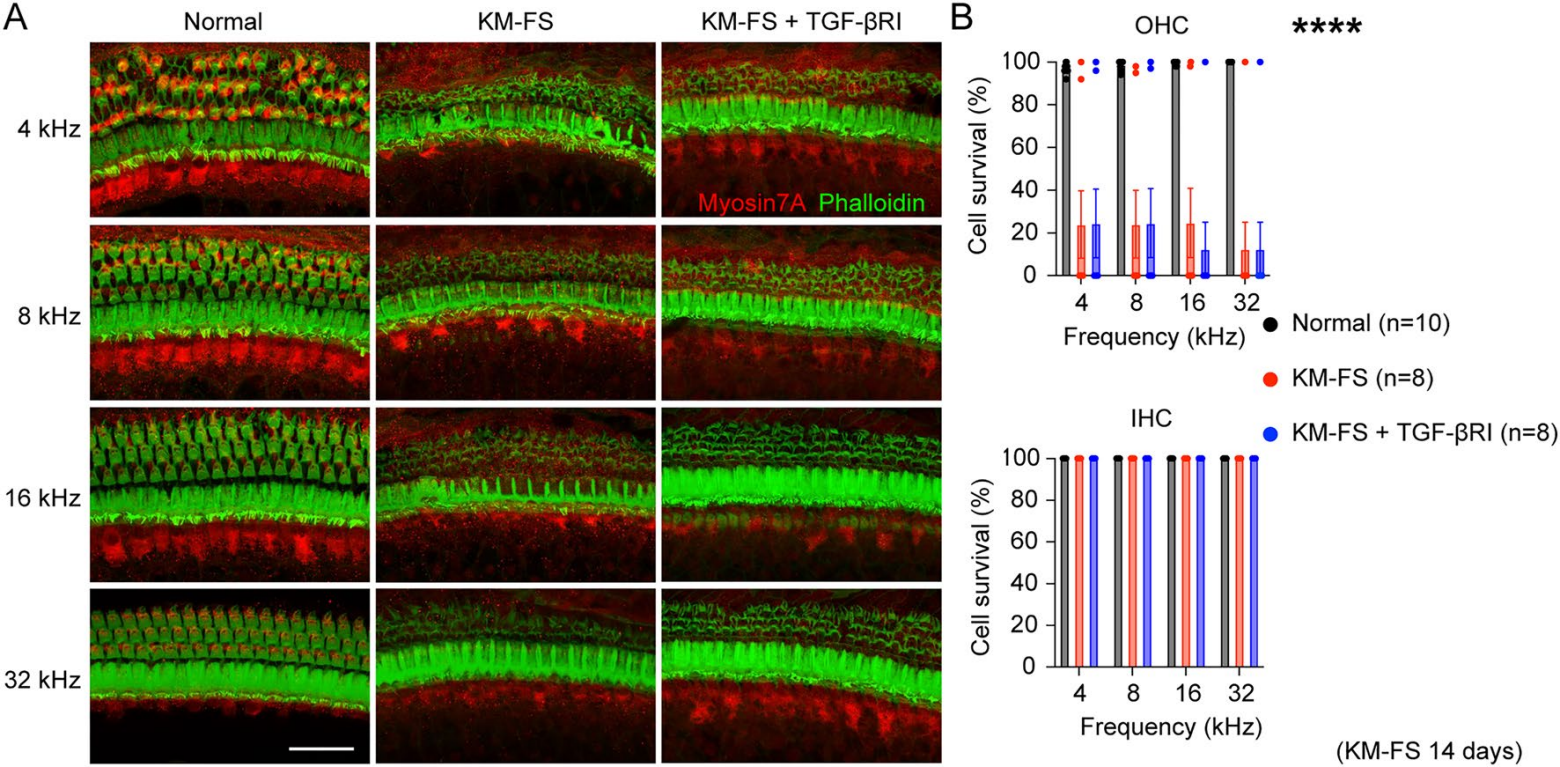
Assessment of hair cell survival. (**A**) Representative immunostaining images of Myosin7A and Phalloidin-iFluor 488 Reagent in the cochlea 14 days after KM-FS administration. Scale bar indicates 20 μm. In the normal group, one row of IHCs and three rows of OHCs were observed, whereas massive OHC loss was observed in the KM-FS and KM-FS + TGF-βRI groups. (**B**) Quantitative OHC and IHC survival rates across groups. OHC survival was significantly lower in the KM-FS and KM-FS + TGF-βRI groups compared to that in the normal group. No significant differences were observed between the KM-FS and KM-FS + TGF-βRI groups. FS, furosemide; OHC, outer hair cell; IHC, inner hair cell; KM, kanamycin; TGF-βRI, TGF-β receptor inhibitor. ****p < 0.0001.

Aligned rows of OHCs (three) and IHCs (one) were observed in the normal group, while a significant loss of OHCs was observed in the KM-FS and KM-FS + TGF-βRI groups (Fig. 4A). For OHCs, the survival rate was significantly lower in the KM-FS (n = 8 ears) and KM-FS + TGF-βRI groups (n = 8 ears) compared to that in the normal group (n = 10 ears) (two-way ANOVA, KM- FS *vs.* normal, *p* < 0.0001; KM-FS + TGF-βRI *vs.* normal, *p* < 0.0001). In contrast, no significant differences were observed between the KM-FS and KM-FS + TGF-βRI groups (two-way ANOVA, KM-FS *vs.* KM-FS + TGF-βRI, *p* = 0.93; Fig. 4B). For IHCs, no significant loss was observed in any group, suggesting their relative resistance to KM-FS-induced ototoxicity. These results are consistent with our ABR results, indicating that topical TGF-βRI administration had minimal effect on ototoxic OHC degeneration.

### Assessment of SGN survival

Studies have shown that KM-FS-induced damage can also affect SGNs, thus SGN survival was evaluated using β-tubulin immunostaining. Evaluations were performed at 28 days after KM-FS administration, following previous reports of significant SGN degeneration at this time point^20^.

**Figure 5:**
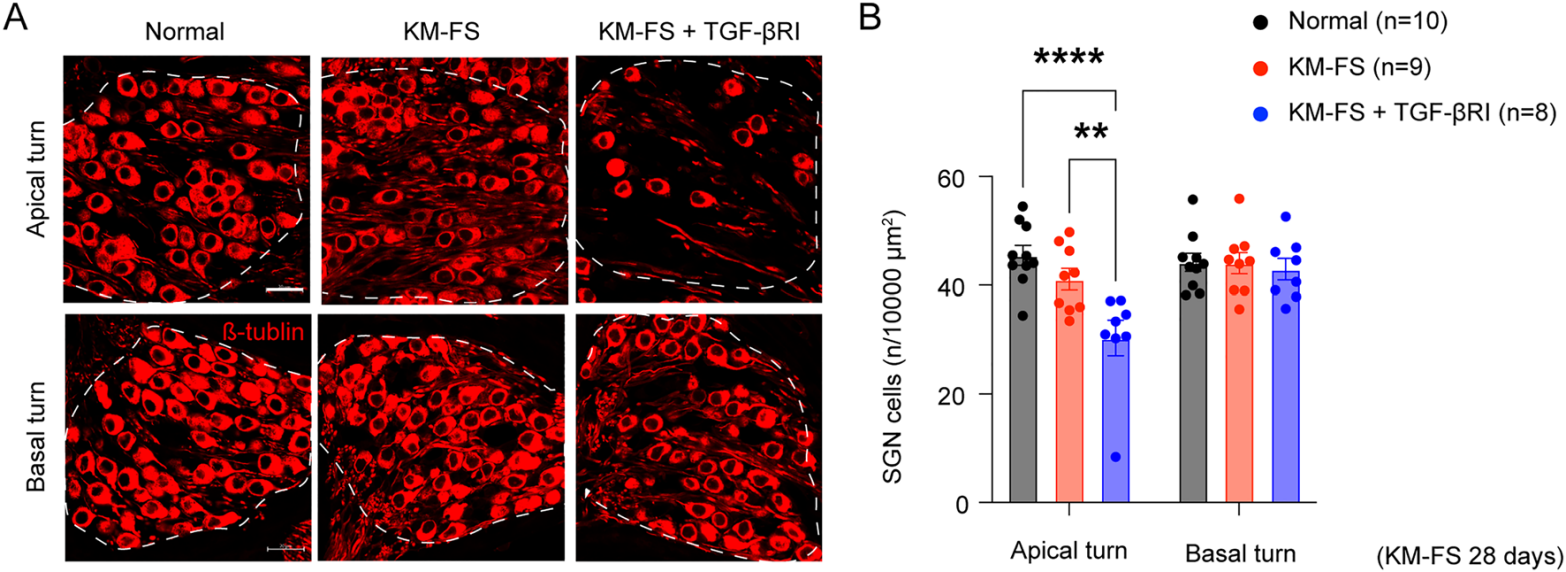
Immunohistochemistry of SGNs and quantitative assessment of SGN survival. (**A**) Representative images of the SGNs. Neuronal cell bodies filled the ganglion in the normal group, whereas SGN degeneration was apparent in the cochlear apical turns of the KM-FS + TGF-βRI group. The dotted line indicates the SGN of Rosenthal’s canal. Scale bar indicates 20 μm. (**B**) In the cochlear apical turn, the number of SGN cells was significantly lower in the KM-FS + TGF-βRI group compared to those in the normal and KM-FS groups. FS, furosemide; KM, kanamycin; SGN, spiral ganglion neuron; TGF-βRI, TGF-β receptor inhibitor. ****p < 0.0001, **p < 0.01.

In the normal group, abundant neuronal cell bodies were observed in both the apical and basal turns (Fig. 5A). However, moderate SGN loss was observed in the KM-FS group, and severe SGN loss was observed in the apical turns of the KM-FS + TGF-βRI group. Quantitative assessment confirmed these findings, revealing that SGN survival in the apical turns was significantly lower in the KM-FS + TGF-βRI group (n = 8 ears) compared to those in the normal (n = 10 ears) and the KM-FS groups (n = 9 ears) (two-way ANOVA, KM-FS + TGF-βRI *vs.* normal, *p* < 0.0001; KM-FS + TGF-βRI *vs.* KM-FS, *p* = 0.003; Fig. 5B). Meanwhile, no significant differences in the basal turns were observed across groups (*p* > 0.05). These results suggest that topical TGF-βRI administration exacerbated KM-FS-induced SGN degeneration in low frequency regions.

### IBA1 infiltration into SGNs

KM-FS ototoxicity is known to trigger immune and inflammatory responses, including macrophage infiltration into SGNs^20,21^. Given the role of TGF-β as an anti-inflammatory agent^22^, we speculated that TGF-βRI might exacerbate cochlear inflammation and SGN degeneration. To investigate this, we utilized IBA1 immunostaining for assessment of macrophage and inflammatory cell infiltration in the cochlea after KM-FS administration^23^. Evaluations were performed at 7 days after KM-FS administration, following a previous study that showed peak macrophage infiltration at 14 days after cochlear damage^24^.

**Figure 6:**
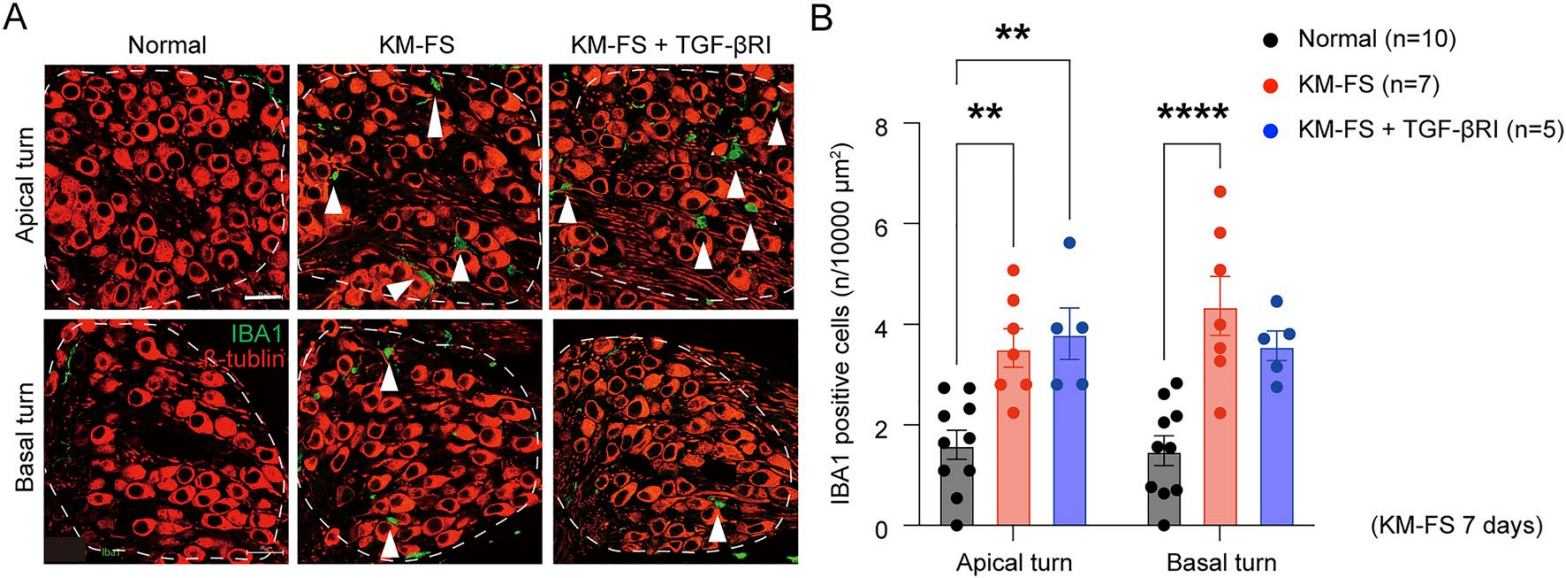
IBA1 immunostaining in SGNs and quantitative assessment of IBA1-positive cells. (**A**) Representative images of SGNs with IBA1 immunostaining. Abundant IBA1-positive cells were observed in the apical turns of the KM-FS + TGF-βRI group. Arrows indicate IBA1-positive cells in the SGN. Scale bar indicates 20 μm. (**B**) The number of IBA1-positive cells was significantly higher in the KM-FS group than that in the normal group for both apical and basal turns. The number of IBA1-positive cells were significantly higher in the KM-FS + TGF-βRI group. FS, furosemide; KM, kanamycin; SGN, spiral ganglion neuron; TGF-βRI, TGF-β receptor inhibitor. **p < 0.01; ****p < 0.001.

The number of IBA1-positive cells in SGNs increased in the KM-FS (n = 7 ears) and KM-FS + TGF-βRI groups (n = 5 ears) compared to that in the normal group (n = 10 ears) (Fig. 6A). Quantitative evaluation confirmed this finding, showing that the number of IBA1-positive cells in apical and basal turns was significantly increased in the KM-FS and KM-FS + TGF-βRI groups compared to that in the normal group (two-way ANOVA, apical turn, KM-FS *vs.* normal, *p* = 0.002; KM-FS + TGF-βRI *vs*. normal, *p* = 0.002; basal turn, KM-FS *vs.* normal, *p* < 0.0001, Fig. 6B). Although no significant differences were observed between the KM-FS + TGF-βRI and KM-FS groups, the KM-FS + TGF-βRI group demonstrated higher IBA1-positive cell numbers in the apical turns, which was also where the significant loss of SGNs was observed. These results suggested that macrophage and inflammatory cell infiltration induced by KM-FS ototoxicity may be activated by topical TGF-βRI administration, resulting in severe SGN degeneration.

### Cleaved Caspase-3 expression in SGNs

Alongside macrophage infiltration and tissue inflammation, apoptosis has been implicated as a mechanism of SGN degeneration after KM-FS administration^25^. Given the role of TGF-β signaling in apoptosis^26^, we investigated SGN apoptosis using immunostaining for cleaved Caspase-3. Evaluations were performed at 7 days after KM-FS administration, following previous reports that showed increased SGN degeneration apoptosis from 7 to 28 days after administration^27^.

**Figure 7:**
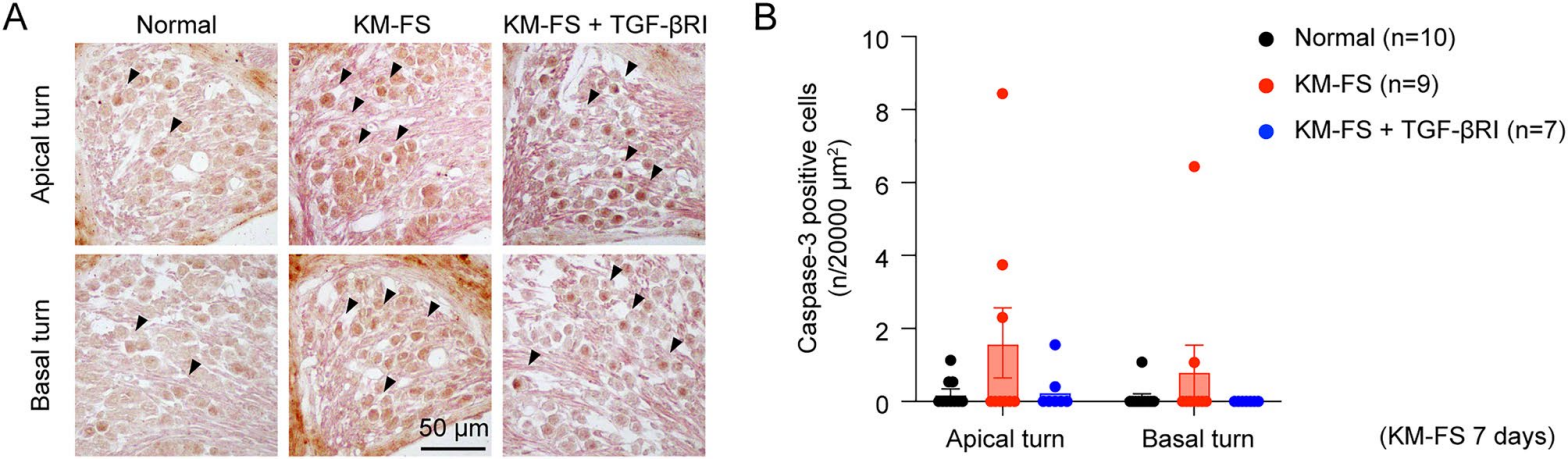
Cleaved Caspase-3 expression in SGNs. (**A**) Representative immunostaining image of cleaved Caspase-3 in SGNs 7 days after KM-FS administration. A small number of positive cells were observed in the KM-FS group, whereas few numbers were observed in the normal group. Scale bar indicates 50 μm. (**B**) No significant differences in the number of cleaved Caspase-3-positive cells were observed across groups. FS, furosemide; KM, kanamycin; SGN, spiral ganglion neuron; TGF-βRI, TGF-β receptor inhibitor.

Increased cleaved Caspase-3-positive cells were observed in the KM-FS treatment group (Fig. 7A). However, quantitative assessment revealed no significant differences across groups (two-way ANOVA, *p* > 0.05, Fig. 7B), suggesting that apoptosis might not be involved in the severity of SGN loss caused by TGF-βRI administration.

## Discussion

In this study, topical TGF-βRI application to the inner ear of KM-FS-induced ototoxicity mice models resulted in exacerbated SGN degeneration in the cochlear apical turns. Increased macrophage infiltration was observed in SGNs after KM-FS administration, suggesting the promotion of cochlear degeneration by an active inflammatory response. Furthermore, TGF-β signaling might have a protective role in KM-FS-induced SGN degeneration, since suppressed signaling was shown to exacerbate SGN degeneration.

The KM-FS model has been extensively studied for its ability to induce cochlear damage, resulting in HC and SGN degeneration^28,29^. Following systemic administration, KM rapidly reaches the OC via the stria vascularis, where it is internalized by HCs through the MET channel. This uptake triggers the production of ROS and free radicals, thereby causing cochlear degeneration. Moreover, ototoxic damage was found to exhibit a gradient of increasing HC susceptibility from the apex to the base, corresponding to the transduction currents in the cochlea which are larger in the basal than in the apical OHCs and further reduced in IHC^7^. Studies have thus proposed possible mechanisms for ototoxicity-induced SGN degeneration, such as secondary changes following HC degeneration and ROS-induced apoptosis^30^. However, the underlying mechanisms remain unknown. Approximately 90–95% of SGNs are type I SGNs, which make a single bouton connection with IHCs^31^, unmyelinated type II afferents can survive cochlear damage^32^. A comparison of neurofilament and peripherin labeling of auditory nerve fibers and SGN in cochlear cultures revealed that peripherin-positive type II fibers are more resistant to KM damage than type I fibers^2^. SGN degeneration by KM administration could be both type 1 loss by secondary SGN degeneration due to OHC loss and type 1 primary degeneration induced even if IHCs survive.

In the present study, OHC and SGN degeneration was observed in the acute phase of ototoxicity, resulting in significant deafness within 1 month after KM-FS administration. While convenient for basic inner ear research due to its ease of induction and versatility^4^, this KM-FS mouse model requires careful husbandry and observation, since increasing KM-FS doses can easily result in death. Substantial inter-individual differences in the degree of induced cochlear damage have been documented in previous reports^16^, which are consistent with our observation of OHC survival. Additionally, C57/BL6 mice in this study exhibited lesser HC losses and reduced hearing impairment compared to CBA and BALB mice following KM administration^33^.

TGF-β is a cytokine involved in multiple biological processes, including cell proliferation, differentiation, and inflammation, and has been implicated in many diseases, such as atherosclerosis and fibrotic diseases^34^. Moreover, clinical studies have investigated the potential of TGF-β signaling inhibitors as anticancer agents^35^, and their efficacy has been evaluated in chronic liver and kidney disorders^36,37^. In the context of cochlear damage, elevated TGF-β expression levels have been observed after acoustic overstimulation. TGF-β inhibitors in these cases suppressed cytokine and NADPH oxidase production, resulting in the attenuation of hearing loss^15^. However, some studies have reported the opposite; adenoviral vectors encoding the TGF-β gene were injected into the cochlea of guinea pigs to overexpress TGF-β, thereby protecting HC and hearing from KM-induced inner ear damage^38^. As such, the detailed role of cochlear TGF-β signaling remains unclear.

Our findings showed that TGF-β receptor expression was prominent in the cochlear LW, OC, and SGN. Notably, significant elevations of TGF-β receptor expression was only observed in SGN, which was suppressed with topical TGF-βRI application (Fig. 2D). This is consistent with studies in other organs, where increased TGF-β receptor expression was observed in damaged liver tissue, and its suppression reduced liver damage in mice^39^. Furthermore, TGF-β signaling increases its receptor expression on the cell surface, as well as enhances its own signaling responses, indicating the complex biological interactions between TGF-β and TGF-β receptors.

SB431542, the TGF-βRI used in this study, has been shown to prevent TGF-β-induced Smad2 and Smad3 activation without affecting cell surface receptor levels^40^. Although KM-FS administration did not significantly affect TGF-β expression in this study, it is possible that the increased receptor levels may have modulated TGF-β signaling responsiveness. TGF-βRI administration has been thought to suppress TGF-β receptor expression and inhibit TGF-β signaling through feedback mechanisms^41^. However, our study showed that SGN degeneration was significantly increased in the cochlear apical turns following TGF-βRI application (Fig. 5B). This seemingly contradictory result may relate to the complex role of macrophages in cochlear damage. Although macrophage infiltration plays a protective role against SGN degeneration^24^, excessive infiltration and inflammatory cytokine production can exacerbate tissue damage^42^. In this study, we observed that macrophage infiltration into the SGNs was induced by KM-FS administration, and TGF-βRI application resulted in increased IBA1 expression and exacerbated SGN degeneration. Macrophage infiltration has shown to be significantly increased in the cochlear apical turns of rats after KM administration, suggesting an active inflammatory response in the apical region^42^. This was consistent with our results of significant SGN degeneration in the apical turns. Another study reported elevated TGF-β mRNA levels and decreased nitric oxide (NO) production in the brain tissue of rats with transient ischemia of the middle cerebral artery. A similar inhibitory effect on NO production was observed in cultured microglial cells of ischemic brain tissue, resulting in sustained inhibition of IκB degradation. This suggests that TGF-β signaling has a sustained anti-inflammatory effect on the microglial cells of ischemic brain tissue^22^. However, our study showed that TGF-β signaling was not involved in apoptosis, despite known studies of apoptosis in SGN degeneration^43,44^.

Cochlear implant therapy has become the primary treatment modality for sensorineural hearing loss in recent years. In cochlear implantation, prolonging SGN survival and ensuring SGN protection are crucial in optimizing postoperative auditory performance. In this study, we showed that TGF-β signaling inhibition promotes apical SGN degeneration in an animal model of ototoxicity. These results may be of clinical significance, suggesting the potential of TGF-β signaling as a novel therapeutic strategy for protecting SGNs from drug-induced ototoxicity.

Despite the insights offered in this study, several limitations must be acknowledged. First, the impact of sex in KM-FS-induced inner ear damage was not investigated^45^. Although male mice were used in this study, future studies should evaluate potential sex differences in the role of TGF-β signaling in ototoxicity. Second, although TGF-βRI was administered immediately after KM-FS treatment, the optimal therapeutic window for modulating TGF-β signaling after cochlear injury remains undefined. Determining this clinically relevant timeframe is crucial for effective TGF-β signaling modulation after cochlear injury. Third, since the effects of TGF-β signaling on inner ear damage were not directly demonstrated, further detailed analysis of the role of TGF-β signaling in inner ear damage is required. Lastly, despite the clinical efficacy and safety of TGF-β inhibitors in multiple clinical trials for solid tumors, they have not yet reached practical application due to the risk of unintentional tumor growth and inflammatory effects^46^. Therefore, considering the topical application of TGF-βRI in this study, further studies should critically evaluate the safety and efficacy of targeted strategies for preventing SGN degeneration, as conducted in our model.

In conclusion, our study demonstrated that topical TGF-βRI application onto the RWM after KM-FS ototoxicity resulted in suppressed cochlear TGF-β signaling and exacerbated SGN degeneration by macrophage infiltration and inflammatory responses. These findings imply that TGF-β signaling may have a protective role in ototoxicity-induced SGN degeneration.

## Methods

### Study approval

All animal experiments were performed in accordance with the guidelines of the Animal Experimentation and Ethics Committee of Kitasato University School of Medicine, and approved by the Kitasato University School of Medicine Animal Experiment Committee (Approval No. 2022035). This study follows ARRIVE guidelines.

### Animals

Eight-week-old C57/BL6 male mice (N = 139) were used in this study. C57/BL6 is the frequently chosen parental strain for transgenic and knockout mice, and their prior use in KM-induced hearing loss studies has been well-documented^16^. To determine the appropriate KM-FS doses for the ototoxicity animal model, 19 mice were administered various concentrations of KM-FS, and their mortality rates and hearing thresholds were investigated. The remaining 120 mice were divided into three groups for further investigation of the role of TGF-β signaling (Fig. 1B).

### Ototoxic drug application and surgical procedure

Mice were intraperitoneally sedated with medetomidine (0.75 mg/kg), midazolam (4 mg/kg), and butorphanol (5 mg/kg). Following general anesthesia, experimental mice received a single intraperitoneal dose of 200 mg/kg KM sulfate (Meiji, Tokyo, Japan), followed by an intraperitoneal dose of 400 mg/Kg FS (400 mg/Kg; Nichiiko, Toyama, Japan) 30 min after. Immediately after FS administration, the left posterior ear was anesthetized with Xylocaine 1% with Epinephrine (Sandoz, Tokyo, Japan). Under a stereomicroscope (Leica S9E, Leica Microsystems, Tokyo, Japan), the skin was incised, the bulla was exposed, and osteotomies were performed to access the RWM. Afterwards, a 1-mm^3^ gelatin sponge (Spongel; LTL Pharma, Tokyo, Japan) soaked in either 3 µL of SB-431542 (TGF-βRI, 10 mM, diluted in DMSO, Cayman Chemical, Michigan, USA) or DMSO (Nacalai tesque, Kyoto, Japan) was placed just above the RWM and closed.

### ELISA

At 3, 7, and 14 days after KM-FS administration, cochleae were dissected from each group, and TGF-β expression was evaluated using the Mouse TGF-β ELISA kit (BMS 608-4, Invitrogen, MA, USA) (normal group; n = 4 ears, KM-FS group; n = 4 ears, KM-FS + TGF-βRI group; n = 4 ears). Whole cochleae were homogenized with RIPA Buffer, and protein was extracted from the supernatant after centrifugation at 10,000 × *g* for 10 min at 4 °C. After diluting 20 μL of the obtained samples with 180 μL of the Assay Buffer, the TGF-β concentration of each sample was determined from the standard curve of the ELISA kit.

### Assessment of TGF-β receptor expression

At 7 days after KM-FS administration, TGF-β receptor expressions in the cochleae of experimental mice were evaluated via immunohistochemistry (normal group; n = 5 ears, KM-FS group; n = 12 ears, KM-FS + TGF-βRI group; n = 5 ears). Following intracardiac perfusion with 4% paraformaldehyde (PFA) in phosphate buffer (PB), cochleae were collected and dissected for assessment. Specifically, the bone near the apex was removed, and 4% PFA was perfused topically through the round and oval windows and fixed overnight. After decalcification in 5% ethylenediaminetetraacetic acid (EDTA) for 1 week, cochleae were embedded in paraffin and sliced to 4-µm-thick sections. Cochlear immunostaining was performed using the anti-TGF-β receptor antibody (ab61213, 1:100, Abcam, Cambridge, UK) as the primary antibody, applied overnight at 4 °C, and DAKO Real Envision (K5007, Dako, CA, USA) as the secondary antibody, applied for 2 h at room temperature. After three washes in PBS, slides were stained with DAB. Semi-quantitative analysis was performed using ImageJ Fiji 1.53c software (National Institutes of Health, Bethesda, MD, USA)^47,48^, where 4–6 sections were stained every 64 μm from the cochlea, selecting the section with the largest SGN area. Cochlear LW, OC, and SGN were then evaluated separately in the apical and basal turn regions. Furthermore, DAB-positive intensity per unit area in the LW and OC and DAB-positive cell area percentage per unit area in the SGN were measured in each group. Expressions in the normal group were used as the standard (converted to 100%), whereas expressions in the KM-FS and KM-FS + TGF-βRI groups were evaluated relative to each other.

### Auditory brainstem response

ABR was performed to measure the hearing thresholds of mice before, 14 days after, and 28 days after KM-FS administration. First, mice were intraperitoneally sedated using medetomidine (0.75 mg/kg), midazolam (4 mg/kg), and butorphanol (5 mg/kg). Measurements were then conducted in a soundproof room, where mice were placed on a warm desk and equipped with subcutaneous needle electrodes over the nose (reference) and mastoid (recording) and a ground electrode on the tail^49,50^. ABR waveforms were recorded using tone burst stimuli at frequencies of 4, 8, 16, and 32 kHz at 5-dB intervals, and a total of 512 responses were averaged using the Neuropack Sigma System (Nihon Kohden, Tokyo, Japan). Hearing thresholds were calculated as the sound level required to produce a voltage response above the mean noise level for each recording.

### Assessment of HCs

At 14 days after KM-FS administration, HC survival was evaluated in the cochleae of the experimental mice (normal group; n = 10 ears, KM-FS group; n = 8 ears, KM-FS + TGF-βRI group; n = 8 ears). Following intracardiac perfusion with 4% PFA in PB, cochleae were removed and fixed in 4% PFA in PB overnight. After decalcification in a 5% EDTA for 1 week, cochleae were dissected by removing the LW and tectorial membranes. OC immunostaining was performed using Myosin7A (ab3481, 1:100, Abcam, Cambridge, UK) as the primary antibody, applied overnight at 4 °C, Alexa Fluor 546 (A-11035, 1:200, Invitrogen, MA, USA) as the secondary antibody, applied for 1 h at room temperature, and Phalloidin-iFluor 488 Reagent (ab176753, 1:500, Abcam, Cambridge, UK) applied for 1 h thereafter. Sections were then enclosed in glass slides and observed under a confocal laser microscope (LSM710; Zeiss, Jena, Germany). The total cochlear length was determined for each cochlea, and a cochlear frequency map was computed to precisely localize the HC from the 4-, 8-, 16-, and 32-kHz regions. OHC and IHC loss was measured in each region, and survival rates were expressed as percentages.

### Assessment of SGN survival, IBA1, and cleaved Caspase-3 expression

At 28 days after KM-FS administration, SGN survival was evaluated in the cochleae of experimental mice (normal group; n = 10 ears, KM-FS group; n = 9 ears, KM-FS + TGF-βRI group; n = 8 ears). Moreover, macrophage infiltration in the SGN was evaluated 7 days after KM-FS administration (normal group; n = 10 ears, KM-FS group; n = 7 ears, KM-FS + TGF-βRI group; n = 5 ears). Both evaluations followed the same preparation procedure. After intracardiac perfusion with 4% PFA in PB, cochleae was removed and fixed in 4% PFA in PB overnight. SGN immunostaining was performed using anti-TUJ1 (MMS-435P, 1:250, BioLegend, CA, USA) as the primary antibody, applied overnight at 4 °C, and Alexa Fluor 546 (A-11035, 1:200, Invitrogen, MA, USA) as the secondary antibody. Sections were then mounted and observed using a confocal laser microscope. As with TGFβ receptor expression evaluation, 4–6 sections were stained every 64 μm from the cochlea, selecting the section with the largest SGN area. To count the number of surviving cells per area, measurements in the cochlear apical and basal turns were conducted using ImageJ.

For macrophage infiltration, SGN immunostaining was performed using anti-IBA1 as the primary antibody, applied overnight at 4 °C, and Alexa Fluor 488 (A-11008, 1:200, Invitrogen, MA, USA) as the secondary antibody. The number of IBA1-positive cells per unit area was measured in the SGNs of the cochlear apical and basal turns.

For apoptosis, SGN immunostaining was performed using cleaved Caspase-3 (#9661, 1:400, Cell Signaling, MA, USA) as the primary antibody, applied overnight at 4 °C, and DAKO Real Envision (K5007, Dako, CA, USA) as the secondary antibody, applied for 1 h at room temperature. After three washes in TBST, slides were stained with DAB, and the section with the largest SGN area was selected. To count the number of cleaved Caspase-3-positive cells per unit area, measurements in the cochlear apical and basal turns were conducted using ImageJ.

### Statistical analysis

Data were expressed as means ± standard error, and distribution normality was assessed using the Shapiro–Wilk test. Normally distributed data were subjected to two-way ANOVA and Tukey’s post-hoc test for multiple comparisons. Meanwhile, non-normally distributed data were subjected to the Kruskal–Wallis test and Dunn’s post-hoc test for multiple comparisons. All statistical analyses were performed using GraphPad Prism 10 (GraphPad Software Inc., La Jolla, CA, USA), and statistical significance was set at *p* < 0.05.

### Supplementary Information

Supplementary Figures.

## Data Availability

The data presented in this study are available on request from the corresponding author.

## Abbreviations

ABR: Auditory brainstem response
FS: Furosemide
HC: Hair cell
IHC: Inner hair cell
KM: Kanamycin
MET: Mechanoelectrical transduction
OHC: Outer hair cell
PB: Phosphate buffer
PFA: Paraformaldehyde
ROS: Reactive oxygen species
RWM: Round window membrane
SGN: Spiral ganglion neuron
TGF-β: Transforming growth factor-β
TGF-βRI: TGF-β receptor inhibitor
LW: Lateral wall
OC: Organ of Corti
