# Supplementary material for: Suppression of the TGF-β signaling exacerbates degeneration of auditory neurons in kanamycin-induced ototoxicity in mice
Source: Sci Rep. 2024 May 13;14:10910. doi: 10.1038/s41598-024-61630-1 (PMC11091189; doi:10.1038/s41598-024-61630-1)
Supplement: Supplementary file 1 — Supplementary Figures. [file 41598_2024_61630_MOESM1_ESM.docx]

**Suppression of the TGF-β signaling exacerbates degeneration of auditory neurons in kanamycin-induced ototoxicity in mice**

Yoshihiro Nitta, Takaomi Kurioka, Sachiyo Mogi, Hajime Sano, Taku Yamashita

**Supplementary Information**

**Supplementary Fig. S1**

**ABR thresholds before KM and FS administration**

**
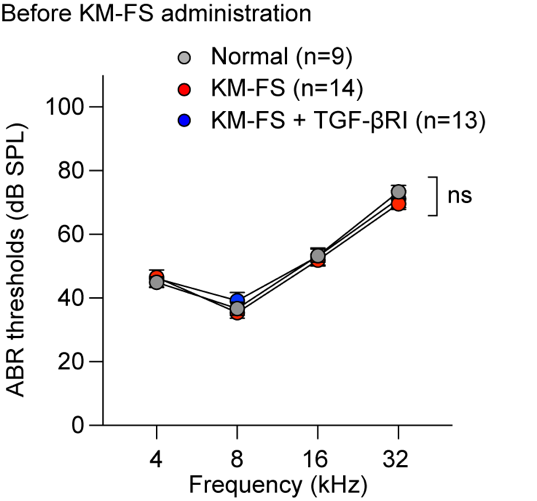
**

ABR thresholds revealed no significant differences among the three groups before KM-FS administration. ABR, auditory brainstem response; FS, furosemide; KM, kanamycin; SPL, sound pressure level; TGF-βRI, TGF-β receptor inhibitor.

**Supplementary Fig. S2**

**Effect of sham surgery on ABR thresholds at 28 days after KM-FS administration**

**
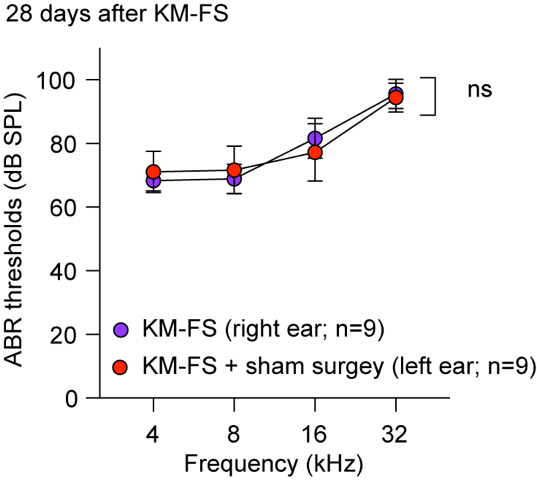
**

No significant difference was observed in ABR thresholds between the sham surgery left ears and non-surgery right ears of KM-FS mice at 28 days after KM-FS administration. ABR, auditory brainstem response; FS, furosemide; KM, kanamycin; SPL, sound pressure level.

**Supplementary Fig. S3**

**Effect of TGF-βRI treatment on contralateral ear in ABR thresholds at 28 days after KM-FS administration**

**
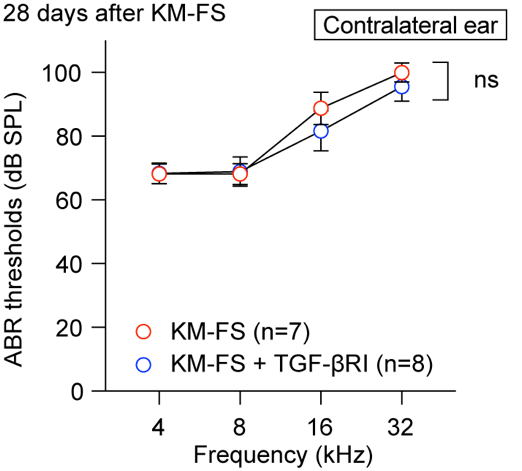
**

There was no significant difference in ABR thresholds in the contralateral ear between the KM-FS and KM-FS+TGF-βRI groups 28 days after ototoxicity. ABR, auditory brainstem response; FS, furosemide; KM, kanamycin; SPL, sound pressure level; TGF-βRI, TGF-β receptor inhibitor.

**Supplementary Fig. S4**

**Evaluation of ABR waveforms and peak 1 amplitude**


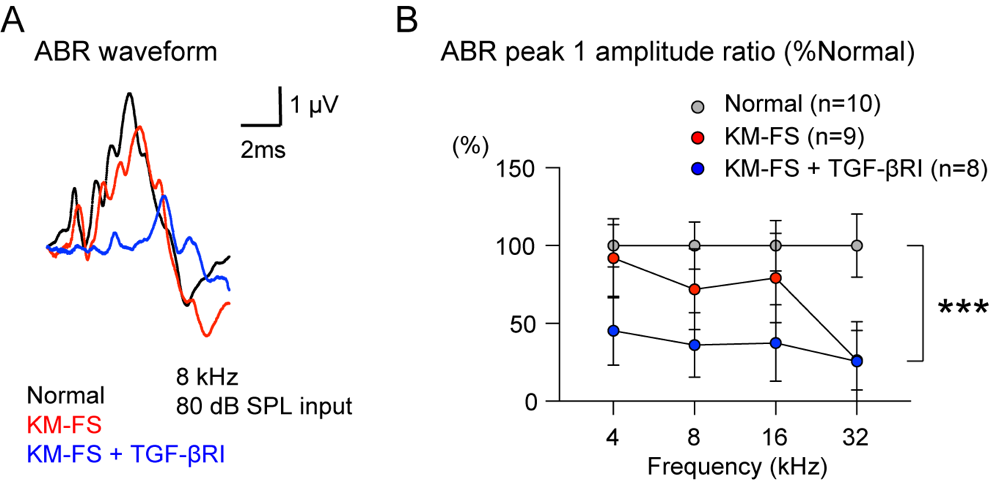


(A) Representative ABR waveforms for each group 28 days after KM-FS administration. (B) Quantitative assessment of ABR peak 1 amplitude in each group. ABR peak 1 amplitudes were normalized to the values in the normal group. The KM-FS and KM-FS+TGF-βRI groups showed decreased ABR peak 1 amplitudes compared to the normal group. ABR, auditory brainstem response; FS, furosemide; KM, kanamycin; TGF-βRI, TGF-β receptor inhibitor.
